# Performance of vision language models for optic disc swelling identification on fundus photographs

**DOI:** 10.3389/fdgth.2025.1660887

**Published:** 2025-08-25

**Authors:** Kelvin Zhenghao Li, Tuyet Thao Nguyen, Heather E. Moss

**Affiliations:** ^1^ Department of Ophthalmology, Stanford University, Palo Alto, CA, United States; ^2^ Department of Ophthalmology, Tan Tock Seng Hospital, Singapore, Singapore; ^3^ Centre of AI in Medicine, Lee Kong Chian School of Medicine, Nanyang Technological University, Singapore, Singapore; ^4^ School of Medicine, University of California Davis, Sacramento, CA, United States; ^5^ Department of Neurology & Neurological Sciences, Stanford University, Palo Alto, CA, United States

**Keywords:** vision language model, disc swelling, papilledema, prompt engineering, artificial intelligence, machine learning

## Abstract

**Introduction:**

Vision language models (VLMs) combine image analysis capabilities with large language models (LLMs). Because of their multimodal capabilities, VLMs offer a clinical advantage over image classification models for the diagnosis of optic disc swelling by allowing a consideration of clinical context. In this study, we compare the performance of non-specialty-trained VLMs with different prompts in the classification of optic disc swelling on fundus photographs.

**Methods:**

A diagnostic test accuracy study was conducted utilizing an open-sourced dataset. Five different prompts (increasing in context) were used with each of five different VLMs (Llama 3.2-vision, LLaVA-Med, LLaVA, GPT-4o, and DeepSeek-4V), resulting in 25 prompt-model pairs. The performance of VLMs in classifying photographs with and without optic disc swelling was measured using Youden's index (YI), F1 score, and accuracy rate.

**Results:**

A total of 779 images of normal optic discs and 295 images of swollen discs were obtained from an open-source image database. Among the 25 prompt-model pairs, valid response rates ranged from 7.8% to 100% (median 93.6%). Diagnostic performance ranged from YI: 0.00 to 0.231 (median 0.042), F1 score: 0.00 to 0.716 (median 0.401), and accuracy rate: 27.5 to 70.5% (median 58.8%). The best-performing prompt-model pair was GPT-4o with role-playing with Chain-of-Thought and few-shot prompting. On average, Llama 3.2-vision performed the best (average YI across prompts 0.181). There was no consistent relationship between the amount of information given in the prompt and the model performance.

**Conclusions:**

Non-specialty-trained VLMs could classify photographs of swollen and normal optic discs better than chance, with performance varying by model. Increasing prompt complexity did not consistently improve performance. Specialty-specific VLMs may be necessary to improve ophthalmic image analysis performance.

## Introduction

Optic disc swelling detection is a critical diagnostic skill in emergent situations. A swollen disc distinguishes serious ophthalmic or neurological conditions from benign ones. Automated methods to achieve this using convolutional neural networks (CNN) with supervised training on fundus photographs have achieved excellent results (area under curve (AUC) 0.96–0.99) (1, 2). However, CNN classifiers are constrained by the input type (photograph) and output of classes on which they are trained (e.g., optic disc swollen vs. normal). Thus, they do not consider other factors that human intelligence considers in arriving at a clinical diagnosis, including patient demographics and history of present illness. Comparatively, generative AI large language models (LLMs) display impressive medical diagnostic performance using text inputs, despite being generalist models without supervised training (3). Vision language models (VLMs) consider images and text as simultaneous input prompts by translating image information into text embeddings. They offer the potential to facilitate interactions between visual representations and language, matching human interpretation of an image more closely than a CNN classifier. In contrast to CNNs for which the input is constrained, VLMs allow for prompt engineering, where input prompts are designed to generate model outputs that accomplish the desired task.

VLMs have potential applications in imaging rich clinical fields such as ophthalmology. The objective of this study is to determine how model choice and prompt impact diagnostic performance for the detection of optic disc swelling on fundus photographs as a first step to understand the capabilities of non-specialty-trained VLMs to address this clinical need.

## Methods

Images of normal and swollen optic discs were obtained from an open-sourced database of images of optic nerve heads (Identification of Pseudo-papilledema, Kaggle) (4). All images were obtained using a non-mydriatic auto fundus camera (AFC-330, Nidek, Japan) (5), downloaded as cropped fundus photographs, with 240 × 240 pixels centered on the optic nerve head in JPEG format. Images “Normal” and “Papilledema” were reviewed by an experienced neuro-ophthalmologist (KL) to verify the labels.

Five VLMs, chosen to represent the spectrum of size and medical training, were used: Llama 3.2-vision (6), LLaVA (7), LLaVA-Med (8), GPT-4o (9), and DeepSeek-4V (10). All models ran locally on an NVIDIA GeForce RTX 4090 GPU, with the models updated to the month of April 2025. Five prompts, with increasing information about the task (Table 1), were developed. Each requested a “yes” or “no” response to the question “does this picture show optic nerve swelling?”. For few-shot prompting, the images were presented in a multiturn protocol.

**Table 1:** Prompts that were used as inputs for the VLMs.

S/N	Prompts	Remarks
1	“Does this picture show optic disc swelling? Reply either yes or no.”	Basic prompt
2	“This fundus photo shows an optic disc. Does this picture show optic disc swelling? Reply either yes or no.”	Context is provided
3	“This fundus photo shows an optic disc. To assess whether an optic nerve is swollen, first assess optic disc margin clarity (sharp/blurred/obscured), then evaluate retinal vessel visibility through disc (clear/partially obscured/fully obscured), and check for peripapillary hemorrhage (present/absent), then synthesize findings to classify if the optic nerve appears to be swollen. Does this picture show optic disc swelling? Reply either yes or no.”	Clinical criteria CoT^a^
4	“This fundus photo shows an optic disc. Compared to reference images: <image> (normal) <image> (swollen). Does this picture show optic disc swelling? Reply either yes or no.”	Few-shot prompting^b^
5	“Your role is a Neuro-ophthalmologist. This fundus photo shows an optic disc. To assess whether an optic nerve is swollen, first assess optic disc margin clarity (sharp/blurred/obscured), then evaluate retinal vessel visibility through disc (clear/partially obscured/fully obscured), and check for peripapillary hemorrhage (present/absent), then synthesize findings to classify if the optic nerve appears to be swollen. You should also compare the reference images: <image> (normal) <image> (swollen). Does this picture show optic disc swelling? Reply either yes or no.”	Role-based with CoT^a^ and few-shot prompting

^a^ CoT prompts instruct the model to perform intermediate steps before arriving at a final output.

^b^ Few-shot prompting is a technique where a language model is given a few examples to guide the model toward a desired output.

A total of 25 diagnostic strategies were defined by all the combinations of VLMs and prompts (prompt-model pairs). To test the performance of each, fundus photographs were presented with the text prompt, and the output was recorded as invalid, correct, or incorrect. Valid output refers to a response that positively or negatively answers the questions. The VLMs are prompted to reply “yes” or “no.” Variations that included punctuations, extra spaces, and phrases like “there is optic disc swelling” are accepted as valid responses. The valid response rate, accuracy rate, F1 score, precision, sensitivity (recall), specificity, and Youden's index (YI) were calculated for each prompt-model pair. To compare between models across prompts, average YI was calculated for prompt-model pairs for each model. Prompt jailbreaking was not attempted and the default and/or recommended temperatures of all VLMs were used to simulate real-world usage. The temperature parameters are given in Table 2.

**Table 2:** Performance of VLM prompt-model combinations for optic disc swelling classification.

Model^a^	Valid response no. (%) (*n* = 1,074)	Accuracy^b^ (%)	Precision^b^ (%)	F1^b^	Sensitivity/recall^b^ (%)	Specificity^b^ (%)	Youden's index^b^
LLaVA-Med (medium open-sourced biomedical model, 7.6 billion parameters, temperature = 0)							
Prompt 1	12 (2.0)	90.5	0.0	0.000	0.0	100.0	0.000
Prompt 2	28 (2.6)	60.7	0.0	0.000	0.0	100.0	0.000
Prompt 3	228 (21.2)	79.8	33.3	0.042	2.2	98.9	0.011
Prompt 4	1,074 (100.0)	36.8	27.1	0.401	77.0	21.6	0.016
Prompt 5	1,074 (100.0)	46.2	28.5	0.393	63.4	39.7	0.031
Average	483 (45.2)	62.8	17.8	0.167	28.5	72.0	0.012
LLaVA (medium open-sourced non-medical model, 7.1 billion parameters, temperature = 0.2)							
Prompt 1	1,074 (100.0)	27.5	27.5	0.431	100.0	0.0	0.000
Prompt 2	1,074 (100.0)	27.5	27.5	0.431	100.0	0.0	0.000
Prompt 3	1,074 (100.0)	27.5	27.5	0.431	100.0	0.0	0.000
Prompt 4	1,074 (100.0)	27.5	27.5	0.431	100.0	0.0	0.000
Prompt 5	734 (68.3)	36.0	20.6	0.329	81.6	25.1	0.067
Average	1,006 (93.7)	27.2	26.1	0.411	96.3	5.0	0.013
GPT-4o (ultralarge closed-sourced non-medical model, undisclosed number of parameters, but purportedly estimated to be more than 200 billion, temperature = 1.0)							
Prompt 1	145 (13.5)	57.2	55.7	0.716	100.0	7.5	0.075
Prompt 2	205 (19.1)	53.7	52.8	0.684	97.2	7.1	0.043
Prompt 3	523 (48.7)	34.4	30.9	0.472	99.4	7.3	0.067
Prompt 4	184 (17.1)	48.9	47.4	0.639	97.7	7.1	0.048
Prompt 5	477 (44.4)	45.5	33.0	0.492	96.9	26.2	0.231
Average	307 (28.6)	47.9	44.0	0.601	98.2	11.0	0.093
DeepSeek-VL2 (large open-sourced non-medical model, 27.5 billion parameters, temperature = 0.1)							
Prompt 1	1,074 (100.0)	69.0	39.4	0.300	24.1	86.0	0.101
Prompt 2	1,074 (100.0)	72.4	48.7	0.114	6.4	97.4	0.038
Prompt 3	1,074 (100.0)	72.5	0.0	0.000	0.0	100.0	0.000
Prompt 4	1,074 (100.0)	72.7	52.8	0.115	6.4	97.8	0.042
Prompt 5	1,074 (100.0)	72.5	0.0	0.000	0.0	100.0	0.000
Average	1,074 (100.0)	71.8	28.2	0.106	7.4	96.2	0.036
Llama 3.2-vision (large open-sourced non-medical model, 88.6 billion parameters, temperature = 0.7)							
Prompt 1	1,069 (99.5)	60.0	37.0	0.470	64.4	58.3	0.227
Prompt 2	1,074 (100.0)	71.0	43.0	0.240	64.7	91.7	0.226
Prompt 3	1,043 (97.1)	59.8	37.4	0.474	63.4	57.9	0.204
Prompt 4	1,072 (99.8)	73.3	53.3	0.338	24.8	91.8	0.166
Prompt 5	1,057 (98.4)	58.8	35.5	0.455	16.6	57.0	0.083
Average	1,063 (99.0)	64.6	41.2	0.395	46.8	71.3	0.181

^a^ See Table 1 for prompt details.

^b^ Metrics calculations are for valid responses only.

## Results

A total of 779 images of normal optic discs and 295 images of swollen discs generated 5,370 inputs for each model. Of the five models, only DeepSeek-VL2 returned valid outputs (yes or no) for all inputs. GPT-4o gave the least number of valid outputs (1,534/5,370, 28.6%). This was followed by LLaVA-Med (45.2%), LLaVA (93.7%), and Llama 3.2-vision (99.0%).

There were eight prompt-model pairs with zero discrimination between the classes (i.e., all valid responses were the same). Four of them (LLaVA-Med with basic prompt, LLaVA-Med with context prompt, DeepSeek-VL2 with clinical Chain-of-Thought (CoT), and DeepSeek-VL2 with role-playing) output “no” for all valid outputs (100% specific, 0% sensitive). Four other prompt-model pairs (the LLaVA model used in conjunction with basic, context, clinical CoT, and few-shot prompts) output “yes” for all valid outputs (0% specific, 100% sensitive).

**Figure 1:**
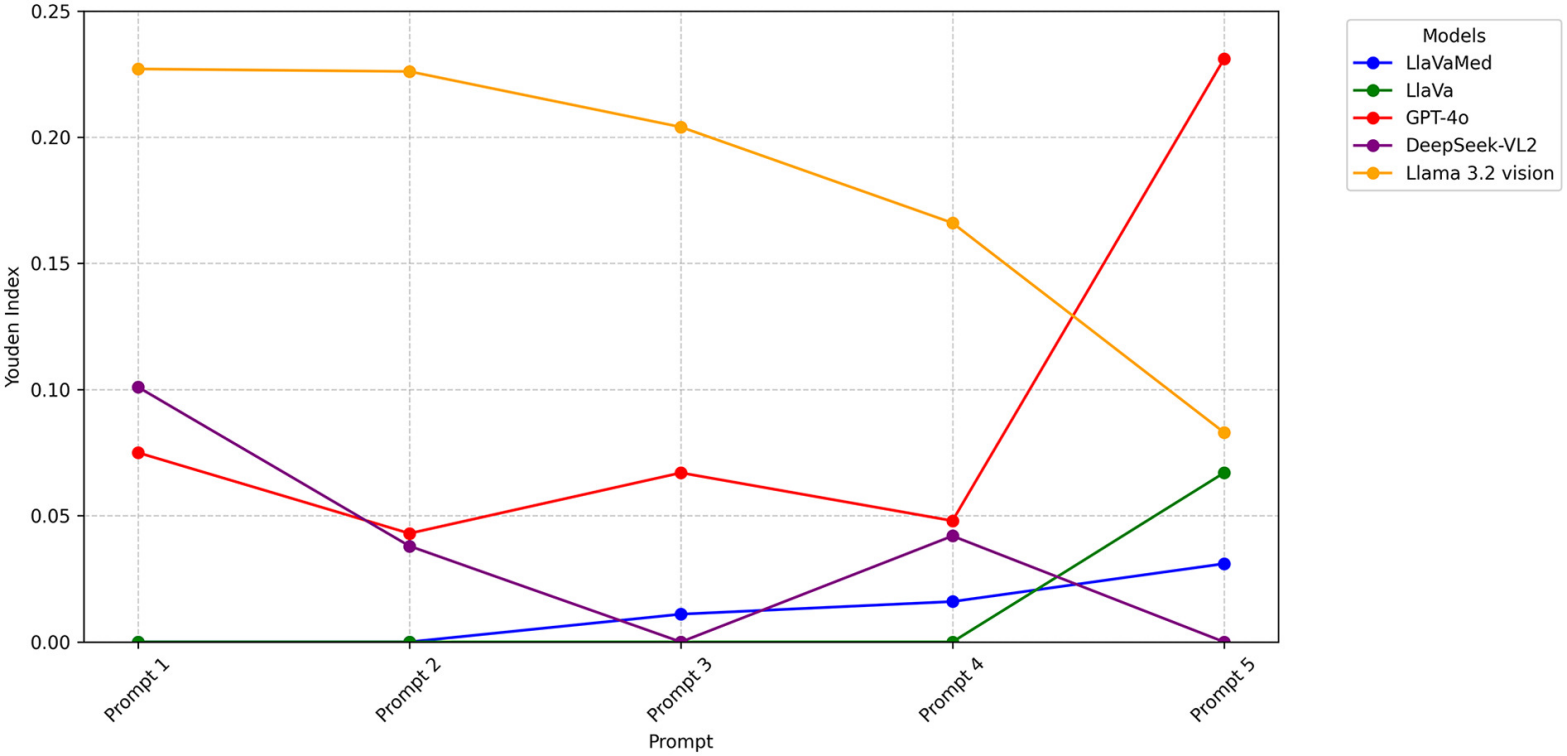
Performance of VLMs and prompts for classification of optic disc swelling photographs. Performance of VLMs and prompts for the classification of 1,074 photographs of optic discs as swollen or not swollen. Youden's index balances sensitivity and specificity, with 0 being chance and >0 being better than chance. Prompts are given in Table 1. Series represent different VLMs. While YI improved with stronger prompts in the case of LLaVA-Med, the opposite trend was observed in Llama 3.2 vision.

Metrics for prompt-model pair performance varied widely (F1 score range 0.00–0.716, median 0.401, accuracy range 27.5%–90.5%, median 58.8%, sensitivity range 0.0%–100.0%, median 64.4%, specificity range 0.00%–100.0%, median 57.0%). Based on YI, calculated to balance between sensitivity and specificity, the best-performing prompt-model pair was GPT-4o with role-playing (YI: 0.231). The results are summarized in Table 2. There was no consistent trend between the amount of information given in the prompt and the subsequent performance measured (Figure 1).

Llama 3.2-vision performed the best across all prompts (average YI 0.181). This was followed by GPT-4o (0.093), DeepSeek-VL2 (0.036), LLaVA (0.013), and, lastly, LLaVA-Med (0.012).

## Discussion

VLMs offer potential advantages over one-pass image classification CNN models for clinical applications because they allow for back-and-forth interaction via multimodal inputs, are generalist, and enable natural interpretability and explainability. This study aimed to benchmark the classification performance of five selected VLMs using five prompts with increasing information on a dataset of 1,074 images of swollen and non-swollen optic discs. The results demonstrate that non-specialty-trained VLMs can perform an optic disc swelling classification with prompt engineering although without supervised training with a YI of up to 0.231. This builds on prior studies focused on retinal pathology, with one reporting 1/69 (1.4%) accurate diagnosis using the image alone (11) and another reporting diagnostic accuracy rates of 64% and 36%, for ChatGPT-4o and Gemini Advanced, respectively (12).

The current iteration of VLM classification of optic disc swelling on fundus photographs is better than chance (YI > 0) but far worse than CNN classifier models on a similar task. Prior groups that performed optic disc classification using machine learning techniques generally achieved an accuracy rate of above 90%. The accuracy rates are as follows: Ahn J et al. achieved 95.89%–98.63%; Milea et al. achieved 91.8%–94.5%; and Szanto et al. achieved 93.6%–96.2% (1, 2, 5). With regard to the performance of VLMs, the reduction in performance is not balanced by the other potential advantages of VLMs and therefore the models tested are not candidates for clinical decision support applications at this time. While prompt engineering impacted performance, there was no consistent relationship between prompts providing increased information about the task and its subsequent performance. These prompting strategies were developed based on their use in LLMs, and it was therefore noteworthy to see whether a similar logic applied to VLMs (12). It is intriguing that the choice of the VLM model had more impact on performance. In a paper by Liu et al., it was found that while longer prompts generally enhance model performance, LLMs still struggle with challenging domain-specific tasks. Specifically, in the realm of disease detection (extracting abnormal findings from radiology reports), a longer prompt, defined as containing at least 200% tokens of the default prompt and providing background knowledge, only had minimal improvements over the default (13). Furthermore, Zheng et al. explored several potential mechanisms on why adding personas may not necessarily improve performances. These include mechanisms like prompt-question similarity and prompt perplexity, the latter referring to the overall probability of a piece of text for a given language model (i.e., lower perplexity indicating more common sequences). The results were inconclusive, with the authors noting that the impact of prompt perplexity was model-dependent (14). Another surprising finding from our study was that model parameter size was not a major contributor to its performance and general medical fine-tuning (LLaVA-Med) did not improve performance compared with the equivalent non-medically tuned model (LLaVA). It will be interesting to note whether model fine-tuning to generate an ophthalmology-specific VLM might improve performance. This would be an area for future research.

Beyond classification performance, other patterns were observed that have important implications for clinical applications. Some prompt-model combinations output a substantial number of invalid outputs despite all prompts attempting to constrain output to “yes” or “no.” Notably, GPT-4o outputs included “I'm unable to analyze medical images for diagnosis. It's best to consult an eye care professional for an evaluation of optic nerve swelling.” While such refusals decrease utility, they also serve as guardrails for areas in which the programmers of the models have determined it has poor performance or risks due to inaccurate output. It is therefore interesting that in up to 48.7% of cases, GPT-4o would still give a response. Notably, this was the case for prompt 3, closely followed by prompt 5 (valid response rate = 44.4%). Clinical CoT formed part of these two prompts, and the use of a reasoning chain could possibly have negated medical advice guardrails set by OpenAI. Another pattern that was seen in this study was that over one-third of prompt-model pairs had zero discrimination, generating a positive or a negative output to all inputs. This occurred primarily with LLaVA-Med and DeepSeek-VL2. These output patterns are concerning because they are difficult to detect based on a single query but negate the utility of the model for the task at hand.

There are a few limitations in this study. First, the use of open-source dataset resulted in the fact that certain clinical details were unknown. In the original paper by Ahn et al. from whence the same dataset originated, cases of optic disc swelling vs. pseudopapilledema were defined separately. Pseudopapilledema was defined as subjects with an elevated optic nerve head and blurred disc margins, with normal visual acuity, visual field, color vision, and pupillary reflex, and who had no changes in their optic nerve head and visual function over a year. These cases of pseudopapilledema were excluded from our study. In addition, as the refractive status of the subjects was not provided, we could not exclude patients with high myopia. Another limitation of our study was that some prompt-model pairs had low valid response rates. This was notably seen in GPT-4o and certain prompt-model pairs in LLaVA-Med. Because the accuracy was calculated based on cases with valid responses, this would have made the model seem more accurate than it is. These limitations may affect the generalizability of the VLM results.

## Conclusion

Using non-specialty-trained VLMs and prompt engineering to classify optic disc swelling on fundus photographs, we compared combinations of five VLMs and five prompts demonstrating performance reaching a YI of 0.231, an F1 score of 0.716, and an accuracy rate of 70.5%. Neither increasing model size nor increasing prompt complexity consistently improved performance. This establishes a baseline performance for current VLM models. Improved ophthalmic image analysis performance is necessary before VLMs can be applied to clinical tasks that combine ophthalmic image and text inputs.

## Data Availability

The raw data supporting the conclusions of this article will be made available by the authors without undue reservation.
